# Developing Sodium Reduction Strategy: Context-Specific Frameworks Targeting Main Dietary Sources

**DOI:** 10.1016/j.advnut.2026.100685

**Published:** 2026-06-30

**Authors:** Puhong Zhang, Feng J He, Guohong Zhang

**Affiliations:** 1 Research and Education Department, Beijing Physical Examination Center, Beijing, China; 2 Food Policy Division, The George Institute for Global Health, Faculty of Medicine, University of New South Wales, Sydney, Australia; 3 Wolfson Institute of Population Health, Queen Mary University of London, London, United Kingdom

**Keywords:** sodium reduction, discretionary sodium, dietary sodium sources, public health policy, implementation roadmap, sodium–potassium ratio

## Abstract

Excessive sodium intake remains a leading global dietary risk. Although global targets exist, progress has been slow, highlighting the need to further align existing strategies with the primary sources of dietary sodium for most of the world’s population. This review aims to support this effort by examining global sodium sources, identifying opportunities to complement current policy approaches, and proposing a strategic roadmap for effective intervention. We classify the 30 most populous countries by their dominant sodium source, revealing that ∼80% of the global population resides in “discretionary-sodium-dominant” countries, where the majority of intake comes from salt and condiments added during cooking or at the table. Together with “discretionary-formula balanced” countries, 90% of the world’s population lives in countries where discretionary sodium plays a major role. Building on existing global guidance that has largely relied on strategies addressing formula sources, we propose a complementary framework for analyzing and reporting sodium sources (natural, formula, and expanded discretionary) to better inform targeted strategies. We outline essential theoretical principles—such as targeting major sources, ensuring structural implementation, and integrating the sodium–potassium balance—and present a concrete, 3-step roadmap for developing national plans. We further propose 9 specific areas for action, emphasizing the need for structured, multisectoral strategies. These include addressing discretionary sodium through mechanisms such as regulated health education for home and restaurant cooks, mandatory training and incentives for food service providers, and scalable digital health programs. We conclude that effective sodium reduction requires context-specific, evidence-based strategies—prioritizing discretionary use where it predominates, and strengthening processed-food-focused approaches where prepackaged foods are the main and growing source.

**Statement of significance**

This work provides the first global classification of nations by their primary dietary sodium sources, showing that current strategies—which prioritize processed food sources—may not fully address the needs of ∼80% of the world’s population living in “discretionary-sodium-dominated” countries. It moves beyond identifying this gap by proposing a novel, actionable policy framework and a structured implementation roadmap specifically designed to develop and deploy mandatory, scalable interventions targeting discretionary salt use in home cooking and food service.

## Introduction

The global mean intake of adults was 4278 mg/day sodium (equivalent to 11 g/day salt) in 2021, far exceeding the WHO recommendation of <2000 mg/d (5 g of salt) [1]. Excessive sodium consumption contributes to 1.7 million annual deaths and is a major driver of noncommunicable diseases [2,3]. Despite the 2013 WHO commitment by Member States to reduce sodium intake by 30% by 2025, progress has been slow, and the target has been extended to 2030 [4].

Dietary sodium sources vary globally. In countries such as Brazil, China, India, and Japan, over half of daily sodium intake comes from discretionary salt added during cooking or at the table. In contrast, in Australia, the United Kingdom, and the United States, daily sodium derives mainly from prepackaged foods (e.g., bread and other bakery products), with discretionary salt accounting for <25% of daily intake [5]. Effective sodium reduction requires targeting both discretionary use and processed foods. Although strategies for prepackaged foods, such as sodium benchmarks and labeling, are well supported, approaches to curb discretionary sodium remain underdeveloped [6,7].

Understanding sodium sources is crucial for developing effective strategies. In China, 90% of dietary sodium comes from discretionary use of salt and high-sodium condiments [8], with 60% from home cooking and 40% from restaurants [9]. Similarly, in India, 90% of sodium intake is from home-prepared food [10], whereas in Japan, >80% comes from home cooking and dining out [11]. In addition, strategies often overlook high-sodium condiments such as soy sauce, monosodium glutamate (MSG), and other seasonings, which contribute substantially to sodium intake in both households and restaurants in these countries [9,12,13].

This paper aims to foster broader awareness and discussion on sodium reduction strategies by identifying limitations in sodium source classification, examining major dietary sodium sources globally, emphasizing the importance of curbing discretionary use, outlining a roadmap for national strategy development, and proposing multisectoral interventions for structured implementation.

## Methods

**FIGURE 1:**
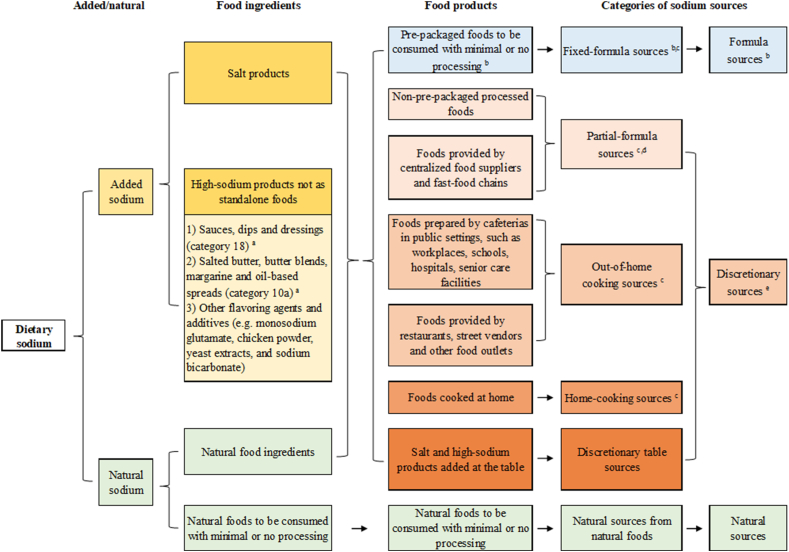
Breakdown and categorization of dietary sodium sources. ^a^The category numbers and definitions follow the WHO global sodium benchmarks (2nd edition) (https://www.who.int/publications/i/item/9789240092013). ^b^Formula sources (fixed-formula sources) refer to prepackaged foods with standard recipes for direct or minimally processed consumption. Excluded are partial-formula food products (see below), as well as food ingredients (e.g., raw meat and vegetables) and high-sodium products (e.g., sauces, dips, dressings) intended for food preparation or not consumed as standalone foods—even if prepackaged. ^c^For practical purposes, naturally occurring sodium is roughly assigned to these categories, because it is difficult to distinguish from laboratory-tested total sodium. ^d^Partial-formula sources refer to food products with relatively stable but nonfixed formulations or lacking standardized nutrition labeling. They are grouped under discretionary sources because fixed-formula strategies (e.g., nutrition labeling, sodium upper limits) alone cannot effectively reduce sodium intake. ^e^Discretionary sources refer to foods prepared with variable recipes, plus salt and high-sodium products added directly at the table—they include all sodium sources except natural foods consumed with minimal or no processing (pure natural sources) and formula sources (see above).

This policy analysis and narrative review was conducted to synthesize evidence and propose a strategic framework for global sodium reduction. Our methodological approach involved 4 key components: *1*) *evidence synthesis*—we reviewed and integrated data from major global reports (e.g., WHO Global Report on Sodium Intake Reduction), systematic reviews, and national studies to establish the current landscape of sodium intake levels, sources, and policy interventions. To this end, we conducted a systematic literature search on predefined themes (e.g., sodium intake level, dietary sodium sources, reduction strategies, interventions, outcome evaluations, and monitoring methods). PubMed, China National Knowledge Infrastructure, and official websites of the WHO and relevant national authorities were searched. No date restrictions were applied, although priority was given to the most recent evidence. Both English and Chinese publications were included. Global reports were primarily sourced from authoritative bodies (e.g., WHO), and national studies were prioritized from peer-reviewed journals and official national reports. *2*) *Development of a novel classification framework for sodium sources*: on the basis of the synthesized evidence, we developed and propose a new conceptual framework for categorizing dietary sodium sources (Figure 1), distinguishing “added” from “natural” sodium in foods, and further subdividing “added sodium” into formula and discretionary sources with contextual details (e.g., stage of addition, food products). *3*) *Country-level analysis and categorization*: applying this framework, we analyzed and categorized the 30 most populous countries—representing 77% of the global population—into 3 groups based on the predominant source (>60% of intake) of dietary sodium: discretionary-sodium-dominated (DSD), discretionary-formula balanced (DFB), and formula-sodium-dominated (FSD). This categorization provides a basis for identifying the key misalignment between major sodium sources and prevailing sodium reduction strategies. *4*) *Recommendations for enhancing global sodium reduction*: drawing on the misalignment analysis and synthesized evidence above, we formulate context-specific recommendations to inform the development and refinement of global and national sodium reduction strategies. A suite of targeted, structured implementation strategies is further proposed to bridge the identified gaps between prevailing policy measures and real-world sodium source profiles.

## Results

### Strategic classification and reporting of sodium sources

To better inform sodium reduction policies, we propose a framework that decomposes and categorizes dietary sodium sources. In this framework, dietary sodium is classified as either natural or added. Added sodium is further stratified across 3 levels: the food ingredient level, the food product level, and the level of sodium source categories based on the degree of formulaic compared with discretionary use (Figure 1).

At the ingredient level, added sodium originates primarily from *salt products* and *high-sodium products* (which are not consumed as standalone foods). Salt products consist predominantly of sodium chloride (NaCl), whereas high-sodium products can be broken down into 3 subcategories: *1*) sauces, dips, and dressings (category 18 in the WHO global sodium benchmark categories); *2*) salted butter, butter blends, margarine, and oil-based spreads (category 10a in the WHO global sodium benchmark categories) [14]; and *3*) other flavoring agents and food additives (e.g., MSG, chicken powder, yeast extracts, sodium bicarbonate)—which represent nonnegligible sodium sources in some regions.

At the food product level, sodium comes from 8 subcategories (quoted and underlined below), which are further grouped into 3 major categories:

1) Formula sources (i.e., fixed-formula sources): “prepackaged foods to be consumed with minimal or no processing,” with standard nutrition labeling for sodium content (e.g., a bag of potato chips or a frozen pizza). Excluded are partial-formula food products (see below), as well as food ingredients (e.g., raw meat, vegetables) and high-sodium products (e.g., sauces, dips, dressings) intended for food preparation or not consumed as standalone foods—even if prepackaged.
2) Discretionary sources: foods prepared with variable recipes (where salt and high-sodium products are added primarily during cooking), plus salt and high-sodium products added directly at the table. We decompose discretionary sources into 4 subcategories: *a*) *Partial-formula sources*, that is, food products with relatively stable but nonfixed formulations or without standardized manufacturer nutrition labeling, including “non-prepackaged processed foods” (e.g., a supermarket’s own-brand roasted chicken) and “foods provided by centralized food suppliers and fast-food chains” (e.g., a fast-food chain hamburger with simple packaging and no standardized sodium nutrition labeling); *b*) *Out-of-home cooking sources*, including “foods prepared by cafeterias in public settings” and “foods provided by restaurants, street vendors, and other food outlets”; *c*) *Home-cooking sources*, that is, “foods cooked at home”; and *d*) *discretionary table sources*, that is, “salt and high-sodium products added at the table.”
3) Natural sources: “natural foods to be consumed with minimal or no processing,” excluding those used as ingredients of food products.

Unlike the conventionally defined “discretionary salt,” which refers only to regular table salt added during cooking or at the table [5,14,15], we propose an expanded concept of “discretionary sodium sources.” This encompasses all sodium sources except fixed-formula sources and pure natural sources, as a substantial proportion of dietary sodium may come from salt and high-sodium products used discretionarily or in variable recipes, and sodium reduction in this context relies primarily on behavior change. For example, a dataset covering 192 restaurants across China indicated that, among added sodium sources in 8131 surveyed dishes, 37% of sodium originated from high-sodium condiments such as soy sauce, sauces and dips, MSG, and chicken powder [13].

In the framework, partial-formula sources are incorporated into discretionary sources to emphasize that strategies targeting fixed-formula sodium sources (e.g., nutrition labeling, sodium upper limits [16,17] may be impractical for real-world implementation because of their unstable recipes or lack of product labeling [18,19]. Furthermore, the impact of reformulating high-sodium condiments may be largely negated by individuals’ discretionary use—that is, using larger quantities than they would with regular products, or adding extra salt [20].

Natural sodium occurs naturally in whole foods that serve either as ingredients (natural food ingredients) or as items consumed with minimal or no processing. Collectively, such sources contribute ∼15% of daily dietary sodium intake [21,22], an amount exceeding the human body’s minimum sodium requirement of 200–500 mg/d [23]. Within the present classification framework, only natural foods eaten with minimal or no processing are defined as natural sodium sources. Naturally occurring sodium inherent in food ingredients is allocated to either formula sources or discretionary sources, given the difficulty of distinguishing natural sodium from laboratory-measured total sodium content. Accordingly, the respective contributions of pure natural sources, formula sources, and discretionary sources aggregate to 100% of total dietary sodium.

In summary, we propose that formula sources be limited exclusively to prepackaged foods intended for direct or minimally processed consumption, whereas all other nonnatural sodium sources be classified as discretionary. Logically, this grouping method will facilitate the advancement of global sodium reduction efforts, particularly in countries where discretionary sources are the primary contributors.

Sodium source analysis and reporting based on a suitable grouping framework should serve as the foundation for a nation to select appropriate reduction strategies—whether behavior-change-based or reformulation-based. Furthermore, it will help inform stakeholders on which strategies to develop or implement, for whom, where, how, and when.

### Major sources of dietary sodium in the world

Guided by the classification framework and sodium source definitions presented in Figure 1, the world’s 30 most populous countries were categorized into 3 groups to inform targeted global sodium reduction efforts (Table 1):

1) DSD countries: >60% of total sodium intake attributable to discretionary sources.
2) DFB countries: 40%–60% of sodium intake from discretionary sources and the remaining 40%–60% from formula sources.
3) FSD countries: >60% of total sodium intake derived from formula sources.

**TABLE 1:** Predominant sodium sources and salt intake levels among the 30 most populous countries.

Population rank/country^1^		WHO region	WB income	Salt intake (g/d)^2^	Main sources of sodium intake^3^	Population-2023	World share (%)	Share in the 30 countries (%)
Discretionary-sodium-dominated countries (>60% of public sodium intake comes from discretionary sources)^4^								
1	India	SEAR	LMC	11.0 [24]	Discretionarily added salt contributed the most to total salt intake, with proportions of 87.7% in South India and 83.5% in North India. 90% of daily salt intake was from the food prepared at home, 7% by packaged foods, and 3% by food prepared outside the home [10,25].	1,428,627,663	17.76	23.08
2	China	WPR	UMC	11.0 [26] 17.7	About 85% of salt intake comes from the salt and salty condiments added during cooking [8,27].	1,425,671,352	17.72	23.03
4	Indonesia	SEAR	LMC	10.5	A significant portion of sodium intake comes from discretionary use, such as adding salt during cooking or at the table [28].	277,534,122	3.45	4.48
5	Pakistan	EMR	LMC	8.9	Majority (71%) use salt in their daily food, whereas more than half also add extra salt at the dining table [29].	240,485,658	2.99	3.88
6	Nigeria	AFR	LMC	10 [30] 6.4	Discretionary use of salt is 82% of total intake, with table salt supplying 78% [31].	223,804,632	2.78	3.62
7	Brazil	AMR	UMC	9	Discretionary salt, coming from table salt and salt-based condiments, is the leading source of dietary sodium, accounting for around 70% of total sodium intake [32,33,34,35].	216,422,446	2.69	3.50
8	Bangladesh	SEAR	LMC	17 [36] 8.9	The estimated 24-h urinary excretion of sodium chloride is 17.0 g/d, with 13.4 g/d from purchased salt [36]. Approximately 80% of the participants added table salt to their meals, although their meals were already cooked and seasoned with salt [37].	172,954,319	2.15	2.79
11	Ethiopia	AFR	LIC	7.1	Both discretionary use and processed foods contribute to sodium intake in Ethiopia; however, discretionary use during cooking or at the table is more prevalent: 80.3% people always add salt during cooking [38]; most households in rural areas do not buy much food from the market [39].	126,527,060	1.57	2.04
12	Japan	WPR	HIC	10.2	52.3% and 57.1% of sodium intake is from discretionary sodium intake by self-cooking, 17.2% and 16.1% from ready-made products, and 30.6% and 26.8% from dining out for men and women, respectively [11].	123,294,513	1.53	1.99
13	Philippines	WPR	LMC	10.4	58% sodium is attributed to added salt in the Philippines [28], and the figure may exceed 60% if the discretionary use of sauces and salt intake by dining out are included.	117,337,368	1.46	1.90
15	DR Congo	AFR	LIC	5.7	No data are available, but generally, in many sub-Saharan African countries, a significant proportion of sodium intake comes from discretionary use of salt during cooking or at the table [40].	102,262,808	1.27	1.65
16	Vietnam	WPR	LMC	10.5	Some 70%−80% of the sodium consumed in the Vietnamese diet comes from salt, fish sauce, and other salty condiments added during cooking or eating [41]. It is also noted that 98% of respondents cook with salt in Vietnam [28].	98,858,950	1.23	1.60
17	Iran	EMR	LMC	9.9 [42] 6.3	Salt sources are the primary contributors to salt intake in Iran. Notably, the discretionary use of added salt, cheese, and salty vegetables alone accounts for a significant portion of salt intake, reaching 57.6% in men and 63.9% in women [43].	89,172,767	1.11	1.44
18	Turkey	EUR	UMC	14.8 [44] 10 [45] 5.3	The majority of salt consumed was from (nonprocessed) bread (34%) and salt added during food preparation (30%), followed by processed foods (21%) and salt added during food consumption (11%) [44,45].	85,816,199	1.07	1.39
20	Thailand	SEAR	UMC	10.8	Sodium intake is mainly attributed to the generous use of condiments and seasonings while cooking and at the table [46]. An early study indicated that ∼90% of dietary sodium comes from condiments such as fish sauce, soy sauce, salt, and seasoning cubes [46].	71,801,279	0.89	1.16
22	Tanzania	AFR	LMC	10.2 [47] 8	In Tanzania, although urban populations have shifted to consuming more processed foods, the 70% rural population likely still relies heavily on discretionary salt use in traditional diets [48].	67,438,106	0.84	1.09
26	Kenya	AFR	LMC	6.1	Discretionary salt intake is a major contributor to salt intake in rural Kenya, but the contribution of salt intake from processed foods is on the rise in urban areas [49].	55,100,586	0.68	0.89
27	Myanmar	SEAR	LMC	10.5	No data available for Myanmar, but the dishes are typically cooked rather than prepackaged [50].	54,577,997	0.68	0.88
29	South Korea	SEAR	LIC	12.3	80% of dietary sodium comes from the discretionary use of kimchi (27%), salt (16%), soy sauce (9%), soybean paste (8%), and other (20%), with the other 20% from processed foods [51].	51,784,059	0.64	0.84
	Subtotal for discretionary-sodium-dominated countries					5,029,471,884	62.51	81.25
Discretionary-formula-balanced countries (40%–60% of sodium intake is from discretionary or formula sources)^4^^,^^5^								
9	Russian Federation	EUR	UMC	9.7	No published data found. Sodium intake in Russia seems to be equally influenced by discretionary use and prepackaged foods, as reflected in the traditional food and diet in Russia [52].	144,444,359	1.80	2.33
10	Mexico	AMR	UMC	8.8	In Mexico, processed and ultraprocessed foods contribute 49% of sodium intake in preschool children, 50% in school-age children, 47% in adolescents, and 39% in adults [53].	128,455,567	1.60	2.07
14	Egypt	EMR	LMC	6.4	Dairy products (660 ± 539 mg/d; white cheese notably, with 464 ± 482 mg/d) and meat, fish, and eggs (424 ± 311 mg/d) were the foods that contributed most to sodium intake among urban Egyptian women [54].	112,716,598	1.40	1.82
24	South Africa	AFR	UMC	6.5	It is estimated that ∼60% of total salt intake is provided from processed foods, whereas the remaining 40% comes from discretionary use of salt in domestic food preparation and salt added to food during meals [55,56].	60,414,495	0.75	0.98
28	Colombia	AMR	UMC	12	The main sources of sodium intake come from both traditional cooking and processed foods, with a major contributor being bakery products (30.5%) [57].	52,085,168	0.65	0.84
30	Uganda	AFR	LIC	7.1	No data published, but home-cooked meals dominate the food consumption [58], which indicates that the dietary sodium intake might be predominantly from discretionary use. However, in Uganda, 8% of households had no salt [59].	48,582,334	0.60	0.78
	Subtotal for discretionary-formula-balanced countries					546,698,521	6.80	8.82
Formula-sodium-dominated countries (>60% population’s sodium intake comes from formula sources)^5^								
3	United States	AMR	HIC	8.9	Processed and prepackaged foods are the major contributors [60]. Sodium added during food manufacturing and processing was the leading source of sodium, accounting for 71% of total sodium intake. Sodium inherent to food was the next highest contributor (14.2%), followed by salt added in home food preparation (5.6%), and salt added to food at the table (4.9%) [61].	339,996,563	4.23	5.49
19	Germany	EUR	HIC	8.7	Processed foods, especially bread, prepared meat products, and dairy products such as cheese, are the primary sources of sodium [62].	83,294,633	1.04	1.35
21	United Kingdom	EUR	HIC	7.1	In the United Kingdom, it is estimated that discretionary salt (added by the individual) accounts for 18% of total intake, 21% comes from sodium naturally present in foods, and ∼61% from sodium in processed foods [63]. In adults aged 19–64 y, white bread, bacon, and ham are together the main contributors of salt in the United Kingdom diet [64].	67,736,802	0.84	1.09
23	France	EUR	HIC	7.6	In France, the main dietary sources of sodium are table salt, condiments, and sauces, as well as processed meats and cheese [65]. Bread, meat products, soups, and cheese are the main contributors to salt intake in the population [66].	64,756,584	0.80	1.05
25	Italy	EUR	HIC	9.7	In Italy, the main sources of sodium intake are cereals (33.2%), meat products (24.5%, especially processed meats), and dairy products (13.6%) [67].	58,870,762	0.73	0.95
	Subtotal for formula-sodium-dominated countries					614,655,344	7.64	9.93
Total						6,190,825,749	76.95	100.00

Abbreviations: AFR, African Region; AMR, Region of the Americas; EMR, Eastern Mediterranean Region; EUR, European Region; HIC, high-income country; LIC, low-income country; LMC, lower-middle-income country; SEAR, South-East Asia Region; UMC, upper-middle-income country; WB, World Bank; WPR, Western Pacific Region.

1 World Bank World Population Prospects: 2022 Revision.

2 If not specified, the data on salt intake levels come from the 2023 WHO global report on sodium intake reduction [6].

3 For countries where no reliable data on sodium sources were found, estimations were made based on published qualitative descriptions and dietary patterns. These countries are DR Congo, Tanzania, Kenya, Myanmar, Russian Federation, Colombia, and Uganda.

4 Discretionary sources refer to foods prepared with variable recipes, plus salt and high-sodium products added directly at the table—they include all sodium sources except natural foods consumed with minimal or no processing (pure natural sources) and formula sources (see below).

5 Formula sources (fixed-formula sources) refer to prepackaged foods with standard recipes for direct or minimally processed consumption. Excluded are partial-formula food products (nonfixed formulations or lacking standardized nutrition labeling), as well as food ingredients (e.g., raw meat and vegetables) and high-sodium products (e.g., sauces, dips, dressings) intended for food preparation or not consumed as standalone foods—even if prepackaged.

These 30 countries cover 77% of the global population, with the vast majority falling into the DSD country category: 19 DSD countries (accounting for 81% of the population within this country sample), alongside 6 DFB countries (9%) and 5 FSD countries (10%). Assuming that other countries not included in the analysis broadly follow this distribution, ∼80% of the global population lives in DSD countries, and 90% of the world’s population lives in DSD or DFB countries, where addressing discretionary sodium is central to strategy development. Among DSD countries, 63% (12/19) have average salt intake exceeding 10 g/d, whereas only 1 across the other 2 groups surpasses this threshold (Table 1).

### Current sodium reduction strategies: progress, gaps, and opportunities for enhancement

The 2016 WHO SHAKE (Surveillance, Harness industry, Adopt standards for labelling and marketing, Knowledge and Environment) package and the 2023 Global Report on Sodium Intake Reduction have laid essential foundations [6,41]. Because most evidence for effective sodium reduction has originated from FSD countries, policies—reformulation, front-of-pack labeling (FOPL), marketing restrictions, taxation—have largely targeted prepackaged foods [6] (Table 2). This works well for FSD countries. However, many DSD countries have adopted similar packaged-food-oriented policies, whereas effective measures for the major sodium sources remain underdeveloped [6,7].

**TABLE 2:** Measures taken worldwide per 2023 WHO global report on sodium reduction^1^

Measures^2^	# (%) of adopted nations	Implementation style	Impacted processes	Main stakeholders targeted^3^	Targeted primary sodium sources^4^
Mass media campaigns	96/194 (49)	Nationwide, in conjunction with other strategies, but with a moderate dose and reach among the general population [7,68]	Food preparation, procurement, and consumption	• Consumers • Cooks • Caterers • Processed food providers • Policymakers	• Discretionary sources • Formula sources
Nutrition labeling (NL): Nutrient declarations	83/194 (43)	Structural implementation, mandatory or voluntary	Food manufacturing and sales	• Processed food providers • Consumers	• Formula sources
Reformulation, such as by setting sodium limits	65/194 (34)	Structural implementation, mandatory or voluntary	Food manufacturing	• Processed food providers	• Formula sources
Public food procurement and service policies	54/194 (28)	Economic impact, for example, taxation, mandatory or voluntary	Food procurement and serving	• Processed food providers	• Formula sources • With potential for discretionary sources
NL: Front-of-pack labeling	40/194 (21)	Structural regulation	Food manufacturing and sales	• Processed food providers • Consumers	• Formula sources
Marketing restrictions	25/194 (13)	Economic impact, for example, taxation Structural regulation	Food procurement and serving	• Processed food providers	• Formula sources • With potential for discretionary sources
NL: Mandatory warning messages on food packages	3/194 (2)	Structural regulation	Food manufacturing and sales	• Processed food providers • Consumers	• Formula sources
NL: Menu labeling	2/196 (1)	Recommendations	Food preparation and sales	• Caterers • Consumers	• Discretionary sources outside the home

1 The WHO global report on sodium intake reduction is available at: https://www.who.int/publications/i/item/9789240069985.

2 Low-sodium salt substitute (LSSS) was only mentioned as an innovation in this report and later received a conditional recommendation by WHO (http://www.ncbi.nlm.nih.gov/books/NBK612023/; https://www.who.int/publications/i/item/SHAKE-the-salt-habit-2nd-ed).

3 Processed food providers include food manufacturers, processors, importers, and retailers.

4 Discretionary sources refer to foods prepared with variable recipes, plus salt and high-sodium products added directly at the table—they include all sodium sources except pure natural sources and formula sources. Formula sources (fixed-formula sources) refer to prepackaged foods with standard recipes for direct or minimally processed consumption.

The revised second edition of SHAKE the Salt Habit (SHAKE v2, 2026) makes important advances, covering food procurement and service policies, lower-sodium salt substitutes (LSSS), a logic model for systematic program design, and explicit advice to tailor interventions to each country’s main sodium sources [4]. These are welcome improvements. Nevertheless, several areas deserve further attention to maximize impact, especially for DSD countries, where 80% of the global population lives.

1) *Best buys still prioritize prepackaged foods.* Under the “best buys” approach [69], prepackaged foods remain a priority in SHAKE v2, where reformulation and FOPL are listed first among its core replicable measures. For DSD countries, investing in nondominant sodium sources risks resistance and low returns if progress on major sources lags. To maximize impact—especially in DSD countries—strategic frameworks should prioritize structural interventions in the food service and household settings, even when local evidence is limited.
2) *Procurement measures require supporting actions.* Removing table salt shakers and high-sodium flavoring agents can face resistance from both food service staff and consumers. Menu labeling and price adjustments rely on stable sodium content and are less feasible when recipes are variable. Therefore, structurally implementing actions targeting both food service personnel and consumers—such as education, training, and certification for staff, along with standardized food service practices to guide and meet consumer demand for lower-sodium meals—could be more feasible, or serve as necessary supportive initiatives from the very beginning.
3) *Sodium–potassium (Na–K) balance is ignored.* SHAKE v2 focuses on sodium and misses the importance of Na–K balance. LSSS is recommended to address discretionary sources in people not at risk of hyperkaliemia; however, LSSS has a modest effect in balancing Na–K intake (Na:K ∼3:1), and widespread use may face cost and safety issues [70,71]. A Na-K balance-centered dietary approach would better support both sodium reduction and potassium enhancement through improvements in ingredient selection, food preparation processes, and food choices—a much safer and more effective way.
4) *Sodium intake monitoring remains difficult.* Twenty-four-hour urine collection is complex; many countries lack reliable data. Spot urinary Na–K ratio offers a practical alternative for population monitoring and intervention guidance, although further consensus is needed.
5) *No framework to classify sodium sources.* SHAKE v2 recommends dietary recalls to identify sources (prepackaged, out-of-home, discretionary) but does not provide a structured framework or definitions. To help countries distinguish formula compared with discretionary sources—and the point and agent of sodium addition—rather than simply reporting by food type, a framework with clear definitions would be helpful.
6) *Structural pathways are critical for behavioral change.* Mandatory progressive food reformulation can reshape public tastes in FSD countries, whereas greater behavioral-change efforts are needed in DSD nations. Although the current intervention package prioritizes mandatory strategies targeting food producers, caterers, and governments, it would benefit from strengthened systematic behavior-change programs at national and regional levels. These programs should be capable of building public knowledge, skills, environments, and social norms to reach nearly all individuals and households, unlike traditional awareness-raising campaigns.

In response, this paper offers concrete suggestions on theoretical considerations, a roadmap for developing a national plan, strategies targeting major sodium sources, and the use of the Na-K balance concept to accelerate sodium reduction.

### Recommendations for enhancing global sodium reduction

To effectively target the primary driver of excess sodium intake, it is essential to establish a robust theoretical foundation, design structured implementation strategies, and foster interdisciplinary and multisector collaboration to ensure coordinated action.

#### Theoretical considerations

Key considerations include:

1) Targeting major sources: effective measures targeting primary dietary sodium sources are a prerequisite for progress; secondary-source interventions are unlikely to succeed if primary sources remain unaddressed. Following Figure 1, a critical first step is to distinguish formula from discretionary sources. For formula sources, key food types and manufacturing enterprises require identification. For discretionary sources, subcategories—partial-formula, out-of-home cooking, home cooking, and table-added sources—must be specified to determine priority intervention settings. In addition, clarifying whether the primary sodium vehicle is regular salt or other high-sodium products is essential for designing educational content and interventions. Priority should be given to developing comprehensive intervention and monitoring frameworks that cover major sodium sources, thereby ensuring effective and scalable implementation.
2) Dealing with nondominant sources: implementing efforts that concurrently target other significant sources of sodium can help create a consistent low-sodium environment and reduce the likelihood of reverting to saltier taste preferences. Where such measures are cost-effective and easy to implement, parallel efforts targeting minor dietary sodium sources are encouraged. However, focusing efforts on minor contributors while devoting insufficient effort and achieving little progress on dominant sources would represent a misallocation of resources and impede scalability.
3) Promoting balanced Na–K intake: Maintaining an optimal Na–K ratio (1:1 molar ratio, as recommended by WHO) serves as a key theoretical foundation and guiding principle for healthy cooking, industrial processing, food choice, and eating. Notably, natural foods typically provide 10–100 times more potassium than sodium [23,72]; however, heavy processing such as blanching, pickling, and stewing with dehydration or draining destroys plant and animal cells, leading to substantial loss of potassium ions naturally present in foods [[73], [74], [75], [76]]. Furthermore, heavy processing often involves adding salt, high-sodium condiments, and sodium-based additives for flavor and preservation. Together, these factors result in a global Na–K intake ratio of 3–5:1 [77,78]. Therefore, promoting natural and minimally processed/refined foods throughout the supply chain is the most effective and safe approach to attaining a balanced dietary Na–K ratio (1:1). In addition, this approach confers multiple independent health benefits, including increasing potassium intake, reducing sodium intake, preserving bioactive nutrients (e.g., vitamins), and avoiding the addition of numerous other potentially health-harmful additives.
4) Structural implementation: Strategies should prioritize broad and long-term population coverage, which can be achieved through top-down regulations, clearly defined objectives, feasible implementation methods, comprehensive intervention content, and reliable evaluation mechanisms to maximize population-level impact. In contrast, interventions relying on one-to-one communication (e.g., dietary counseling), individual pilot projects, and public education campaigns are often limited by narrow coverage or weak impact, and are generally insufficient to effectively reshape the population’s habitual excessive sodium intake [7,79].
5) Avoiding one-size-fits-all evidence extension: Although sodium limits and nutrition labeling have demonstrated effectiveness in lowering sodium levels in prepackaged foods, uncritically extending these same interventions to out-of-home settings—such as restaurant kitchens or consumer meal selection—risks overlooking contextual differences. Evidence supporting their capacity to guide chefs or consumers toward lower-sodium dishes remains unconfirmed, even when applied in restaurant chains [[80], [81], [82], [83]]. More innovative, context-based solutions are therefore needed. For example, encouraging consumers to explicitly request lower-salt options when dining out represents one such alternative approach.

### Proposed roadmap for developing a national plan for sodium reduction

On the basis of the theoretical considerations above, we propose a 3-step roadmap (Table 3) to guide the preparation, development, and implementation of comprehensive sodium reduction programs at the national or regional level. A summary is provided below.

1) *Step 1: baseline investigation and contextual evaluation.* This equips policymakers with foundational data for strategy development and program evaluation. Strategies must be country-specific, ideally targeting the principal sources of sodium intake, as well as the key cooking, manufacturing, and eating practices that either facilitate potassium loss (e.g., through leaching during food preparation or processing) or impede sufficient potassium intake (e.g., by limiting access to or consumption of potassium-rich foods). These strategies should also account for a nation’s gaps in public knowledge and lack of relevant policy frameworks.
2) *Step 2: establishment of measurable, uniform, multisectoral reduction objectives.* This involves setting progressive, feasible targets for the general population and major sodium sources, then allocating them to relevant sectors. Of note, the uniform relative reduction targets for specific sodium sources could be slightly more ambitious than the overall population goal (e.g., a 4% annual reduction, which is higher than WHO’s 30% target for 2013–2025) yet remain feasible—considerably lower than the 1 g/d (≈10%) salt reduction achieved in a 1-y scale-up study [84,85].
3) *Step 3: implementation protocol development.* This produces actionable protocols and measurable standards for all parties to follow.

**TABLE 3:** Three-step roadmap for developing a national sodium reduction plan

Step	Core objective	Key actions
Step 1. Baseline investigation and contextual evaluation		
	Arm policymakers with foundational data to inform strategy development and program evaluation	– Assess population sodium intake and Na–K ratio levels to inform the setting of national targets for reducing sodium intake and increasing potassium intake. – Identify major dietary sodium sources and their contributions using the framework in Figure 1, along with key cooking, manufacturing, and eating practices that promote potassium loss or reduce potassium intake, to guide nation-specific strategies that focus on reducing sodium from primary sources (while covering others) and also improving potassium intake. – Clarify manufacturing, cooking, and eating practices to inform the development of feasible and mutually supported sodium reduction targets and measures targeting different populations, settings, and procedures/timing. – Capture public knowledge, misunderstandings, and misconceptions across diverse populations to facilitate the design of educational and supportive content, activities, and tools for targeted groups. – Conduct contextual, policy, and stakeholder analysis to: (a) map key stakeholders to be engaged and social sources to be leveraged for sodium reduction; and (b) identify the implementation landscape, facilitators, and barriers, and explore solutions.
Step 2. Establishment of measurable, uniform, multisectoral reduction objectives		
	Set progressive, feasible, and measurable targets for the general population and major sodium sources; allocate to relevant sectors	– Adopt a uniform relative reduction target for the overall population and across all major sodium sources (e.g., 4% annually, or 10% over 3 y). – Apply sector-specific metrics, focusing on major sources (sodium concentration/amount or Na–K ratio): • Households: salt or high-sodium product use (weighing method based on dietary survey); spot urine Na–K ratio. • Restaurants or centralized food suppliers: average sodium content of the dishes (whole-food laboratory test, for top 5 selling dishes [86,87]). • Mass caterers: salt or high-sodium product use (business records and warehouse data). • Food manufacturers: product-level sodium content (nutrition labeling information). – Evaluate population reduction using the same sampling and evaluation framework as baseline: 24-h urinary sodium as the reference standard, with multiple spot urine Na–K ratio as an optional method to improve feasibility [88,89].
Step 3. Implementation protocol development		
	Develop actionable protocols and measurable standards for all relevant parties	– Designate a lead coordinating unit. – Identify participating sectors, institutions, and social organizations. – Specify evidence-based, feasible interventions that can be structurally delivered and are optimal for both sodium reduction and potassium increase. – Set a detailed schedule for phased implementation following uniform relative progress across different parties. – Define clear assessment indicators and methods. – Establish regular improvement and coordination mechanisms.

### Proposed strategies for structured implementation

Structured implementation, endorsed by the government, is critical for delivering effective and scalable interventions that influence both individual behaviors and population health. Simply having a national sodium reduction target, general recommendations, demonstration projects, and public awareness campaigns is not sufficient. To address this gap, the following strategic components could be tailored to guide meaningful action.

#### Effectively analyzing and reporting sodium sources

Appropriate analysis and reporting of sodium sources underpin the development of feasible, effective national sodium reduction strategies and policies.

1) For discretionary sources, analyses are more effective when organized by context (e.g., home, restaurants, street vendors), stage of addition (cooking or at the table), and major contributors (e.g., salt, high-sodium condiments). This detailed breakdown informs targeted education and regulatory policies, particularly in DSD countries. However, numerous available studies—even in DSD countries—have tended to analyze and report sodium sources by broad food categories (e.g., condiments, bread products, cereal and grains, meat products, and dairy products) [5,11,25,32,57,90,91], with fewer studies delving into who added what and where [9,10,44].
2) For formula-added sources—particularly in FSD countries—categorization should be further disaggregated by food group (e.g., bread, meat products), specific manufacturer, and manufacturer scale [e.g., small/medium-sized enterprises (SMEs) compared with multinationals]. Such granular categorization enables the identification of key products and manufacturers for effective engagement. For example, the WHO global sodium benchmarks cover 70 food subcategories [14]; setting sodium limits for priority food categories through engagement with major food companies has been successfully implemented in many countries, including the United Kingdom, Australia, and Brazil [33,[92], [93], [94]]; and tailored support for SMEs is necessary to achieve significant reductions (e.g., a 42% salt reduction in a Scottish SME) [94,95]. A global review identified 59 national industry salt reduction programs [96], confirming the value of this categorization approach.

#### Developing a comprehensive and mutually reinforcing strategic framework

A strategic framework should encompass diverse, locally tailored strategies targeting both primary and secondary sodium sources. These strategies must be mutually reinforcing, implemented concurrently, and supported by clear responsibility assignments to government departments and social organizations, along with a robust monitoring system to track progress. This is particularly challenging for DSD countries because such strategies must target a diverse array of actors (e.g., home cooks, restaurant chefs, street food vendors), multiple settings (e.g., households, canteens, market stalls), and varied sodium sources (e.g., table salt, soy sauce, flavor enhancers), and rely predominantly on individual behavior change rather than on legislation.

#### Delivering structured education and communication

We recommend delivering comprehensive education and communication through 4 complementary approaches: *1*) promoting a healthy dietary culture, *2*) developing systematic and population-specific educational materials and models, *3*) leveraging technological platforms for systematic implementation, and *4*) formulating implementation plans to engage stakeholders and reinforce other sodium reduction strategies. Government-led coordination of these initiatives via official health education channels would help ensure extensive coverage and measurable efficacy.

1) Cultivate a healthy dietary culture through sustained media campaigns, such as structurally implemented social marketing and social norm initiatives [97,98]. For example, social marketing campaigns can be designed to model and promote low-salt eating as a socially desirable and normative behavior—using mass media to repeatedly expose target audiences to messages that “most people are choosing less salt” and that “reducing salt is a shared community value.” Specific practices drawn from the literature include framing salt reduction as a positive, achievable goal (e.g., “enjoy flavor with less salt”); using credible local spokespersons (chefs, health workers, community leaders) to demonstrate the behavior; and combining mass media with community-based events (e.g., cooking demonstrations, salt-awareness weeks) to reinforce the social norm [97]. In addition, interventions that provide feedback on how one’s salt intake compares to that of peers (e.g., “you are eating more salt than 80% of people like you”) have been shown to motivate behavior change by leveraging informational eating norms [98].
2) Develop contextualized educational materials grounded in authoritative guidelines and research on dietary sodium sources, barriers to reduction, and evidence-based interventions. These materials should be more targeted, persuasive, and supportive than conventional fact sheets. Emphasis could be placed on several key aspects, including but not limited to the following 2 situations.
  i) Individuals may be hindered by misconceptions about sodium reduction. In China, for example, sodium intake doubles the recommended limit, yet three-quarters of people fear that “less salt means less strength” [99]. Evidence-based clarifications and practical guidance are needed to help convince people that, consuming a typical diet, sodium deficiency is exceedingly rare [1]—a point that may effectively counter commonly held fears.
  ii) Even when a substantial proportion of consumers desire less sodium, the primary remaining barrier may lie with food manufacturers and cooks, who often lag behind. For example, in China, >75% of restaurant consumers find dishes too salty, and ∼50% have even requested less salty options [86]—yet most restaurant owners and chefs continue to favor salty dishes, believing them to be more flavorful [100].
3) Leverage modern social media and artificial intelligence technologies to deliver systematic, personalized health education and facilitate behavior change in daily life [[101], [102], [103]]. Take EduSaltS (School‑based Education Program to Reduce Salt: Scaling‑up in China) as an example [104]. With support and reminders from primary schools based on autogenerated performance evaluations, systematic cartoon-based educational lessons and suggested activities were automatically issued regularly to students’ parents via smartphones. This approach engages the whole family and was found to be effective in reducing sodium intake and blood pressure in randomized controlled trials (RCTs) and subsequent scaling-up studies [85,105,106]. It has the potential to reach the whole population through systematic, long-term education.
4) Develop a comprehensive implementation plan involving key government sectors (e.g., health, education, communication, and market regulation), professional health education institutions, relevant social organizations, and traditional/new social media platforms. The plan should include clear objectives, target populations, planned interventions, supporting materials, and evaluation metrics to effectively promote sodium reduction.

#### Reducing sodium in home cooking

Unlike other settings, home cooks typically lack formal organizations that can influence their behavior or hold them accountable, rendering sodium reduction interventions in this setting particularly challenging. Nevertheless, mounting evidence demonstrates that well-designed education and guidance for home cooks can effectively reduce sodium intake and lower blood pressure, yielding substantial health benefits [[107], [108], [109]].

Effective interventions can be delivered through multiple channels, including schools, communities, primary care facilities, and country-specific women’s or home cook organizations (e.g., the All-China Women’s Federation, India’s National Commission for Women, Brazil’s National Council of Women’s Rights, and the Indonesian Women’s Congress) [[107], [108], [109], [110], [111]]. Schools represent a particularly effective channel. Programs that simultaneously engage both children and their parents—such as the EduSaltS initiative—have yielded sustained reductions in dietary sodium intake and blood pressure [106,112].

Practical measures supported by evidence encompass a range of strategies: educational initiatives; the use of salt-restricted utensils, dosing devices for cooking, and warning labels on salt containers; substitution of regular salt with LSSS; counseling from healthcare professionals; and peer support combined with friendly competitions [107,108,110,113]. In a cluster RCT in China, a community-based intervention package targeting home cooks significantly reduced 24-h urinary sodium excretion and blood pressure [108]. Successful implementation of these measures requires support and oversight from higher-level government or health authorities [107].

#### Reducing sodium from out-of-home cooking sources

Available evidence shows that restaurant and caterer chefs often use more salt and high-sodium products than home cooks, making dishes saltier than what most consumers expect [86,114,115]. For DSD countries, implementing multisector-supported out-of-home cooking interventions—such as training and licensing cooks in sodium reduction techniques [115]—is therefore crucial. Drawing on the Behavior Change Wheel theory [116] and its counterpart within China’s CHRPS (Communication and education, salt reduction in Home cooking, salt reduction in Restaurants, reducing salt content in Pre‑packaged food, and Surveillance and evaluation) strategy [8], a structured strategic framework may include the following components:

1) Launching mass media campaigns to educate the public on healthy diets, encouraging consumers to request less salty dishes when dining out, and promoting this behavior as a social norm (*Guiding demand*).
2) Encouraging and facilitating restaurants to build a healthy image by declaring themselves as “healthy restaurants,” committing to and offering less salty food options and reduced-salt menu choices (e.g., dishes with 50% or 70% less salt), and removing salt shakers from tables (*Tailoring to individual preferences while boosting restaurant patronage*).
3) Train chefs to prepare low-salt dishes and incorporate Na-K balance principles into chef training and licensing requirements. Encourage chefs to cut salt use, for example, by 20%, a reduction that does not compromise sensory taste [117]. Notably, in some contexts, chefs need to recognize that most consumers perceive restaurant foods as saltier than desired—for example, 75% in China [100] (*Empowering chefs with knowledge and skills*).
4) Waitstaff should be trained to recognize that most consumers may prefer less salty foods [100] and to confirm their preferred salt level—along with other dietary inquiries—before submitting the order to the kitchen (*Demonstrating a healthy restaurant culture and respecting consumers’ health preferences*).
5) Engaging academic and industry associations by assigning them clear roles and goals to facilitate the implementation of these actions (*Support from research institutions and professional societies*).

#### Addressing partial-formula sources

As the pace of life accelerates, food sales from quick service restaurants, contract catering companies, and retail food enterprises are increasing [118,119]. The degree of formula fixation for these products lies between home cooking and prepackaged foods. Depending on the context, measures can be adopted that lean either toward those targeting restaurant cooking (e.g., guiding consumer demand for less salt) or toward those targeting prepackaged foods (e.g., setting sodium content limits). Specific approaches primarily include nationally mandated nutrition standards and regulations to reduce sodium in public sector catering and school meal procurement [80,120]; promotional encouragement of procuring lower-sodium products through contractual agreements with institutional meal providers [121]; and consumer-driven approaches, such as app-based requests for less salt when ordering from meal delivery platforms [122].

#### Addressing formula sources

This domain targets prepackaged foods. Numerous mature strategies exist globally, including public health education, support for healthier choices, reformulation with upper sodium limits, FOPL, and regulatory or fiscal policies [14,41,[123], [124], [125], [126]]. However, global reviews indicate that their implementation and evaluation remain limited [6,127]. To advance implementation, the following points merit consideration:

1) In DSD countries, prepackaged food reduction efforts must coordinate with discretionary-source measures and align their targets accordingly. Yet, given rising packaged food consumption and international experience, this sector should not lag behind—provided that targets are regularly evaluated against realistic benchmarks.
2) Priority should be directed toward foods that are major contributors to dietary sodium intake, recognizing that these contributors vary by country and should be identified through baseline assessment.
3) Public awareness and use of nutrition labels remain low [6,128,129]. Strengthened efforts are therefore needed to promote nutrition labeling. Electronic nutrition labeling (e-label) systems—particularly technology-enabled digitalized FOPL—may offer a promising approach to enhance label utility, monitoring, and evaluation [130,131].

#### Prioritizing programs integrated into routine work and targeting diverse sodium sources

Sodium reduction programs that can be delivered in a structured manner and simultaneously target multiple sodium sources are highly preferable, especially those that can be integrated into or serve as a substitute for routine work. The EduSaltS program exemplifies this approach: it delivers interactive learning and practical experience to families through schoolchildren, aiming to reduce sodium intake from home-cooked dishes, out-of-home dining, and prepackaged foods. Furthermore, as a school-based health education system, EduSaltS can accommodate various health education curricula endorsed by governmental authorities. This functionality substantially supports routine school health education mandates and strengthens the program’s scalability [132].

#### Establishing a robust monitoring and evaluation system

Developing robust systems to continuously assess progress and outcomes against established implementation plans and goals is critical to ensuring harmonized progress across different strategies. Core evaluation metrics are outlined in Table 3. The scope, content, and frequency of evaluation should be tailored to national contexts and local implementation arrangements. Recommended assessment intervals include annual reviews of multisectoral task delivery, detailed surveys of sodium levels in home-cooked, out-of-home and prepackaged foods every 2–3 y, and comprehensive reassessments of national population sodium intake targets every 5 y. Interim evaluation findings can further inform targeted strategy adjustments as needed.

### Accelerating sodium reduction using the Na–K balance concept

Although global sodium reduction remains a challenging endeavor, the issue has become such a cliché that it generates little novelty or motivation among stakeholders. A shift in perspective is required to drive progress. Reframing monitoring and intervention toward a Na–K balanced diet may offer a viable solution [133].

#### Utilizing Na–K ratio for monitoring purposes

The 24-h urine collection method, despite being the reference standard, is hampered by low adherence (6%–47% incomplete collections) [134], day-to-day variability (16%–29% coefficients of variation) [135], high participant burden, and operational complexity [136], highlighting its limited practicality for routine population monitoring. These issues partly explain the lack of population sodium intake data in many countries [137]. Although the need for a convenient and accurate surrogate for the 24-h urine method persists, methods based on spot urine samples have been explicitly discouraged by authoritative bodies [138,139]. In this context, the spot urine Na–K ratio—which reflects relative electrolyte balance rather than absolute sodium excretion—offers a practical alternative [88,140]. This possibility is supported by the following observations:

1) *Health benefits*: adequate potassium intake is strongly linked to blood pressure and overall health [[140], [141], [142], [143], [144], [145], [146], [147]]. Increasing potassium intake offers additional cardiovascular benefits beyond sodium reduction [[148], [149], [150], [151], [152], [153], [154], [155], [156], [157]]. Increasing evidence supports an association between urine Na–K ratio and blood pressure [142,145,[158], [159], [160], [161]], as well as the effectiveness of self-monitoring of the Na–K ratio in lowering blood pressure [162].
2) *Gap to guidelines*: the global average Na–K intake ratio is ∼3.24:1, with values exceeding 5 in South and East Asia, significantly exceeding the WHO-recommended ratio of ≤1:1 [6,23,72,77,78,163].
3) *A more reliable dietary metric*: unlike 24-h urine collection-based sodium intake, which correlates with energy intake (*r* > 0.7 for most age/sex groups) [164], the urine Na–K ratio—derived from either spot or 24-h samples—more directly reflects dietary Na–K balance, independent of meal size. This facilitates comparisons across population subgroups differing in sex, age, or occupation, factors that may influence total food (energy) intake. Moreover, at the population level, spot urine Na–K ratios show strong agreement with 24-h urine Na–K ratios (e.g., population-level correlation *r* = 0.96) [140].
4) *Facilitation of sodium reduction*: achieving a balanced Na–K diet requires both sodium reduction and increased potassium intake, with reducing salt addition during food preparation as the major solution [141]. Frequent self-monitoring of the spot urine Na–K ratio can help individuals adhere to an appropriate dietary sodium intake [162,165,166].
5) *Convenience in sampling and testing*: unlike the 24-h urine method, the Na–K ratio uses spot urine samples, which are far easier to obtain and are well-supported for use with repeated samples to manage variability [140,[167], [168], [169], [170]]. Portable testing devices that measure the spot urine Na–K ratio further increase convenience by offering rapid (within 1 min), affordable results [110,171]. A 2025 meta-analysis confirmed that self-monitoring using these devices significantly reduces urinary Na–K ratio, sodium excretion, and systolic blood pressure [162].
6) *Case study*: Japan recommends using averaged spot urine Na–K ratios over 4 d, with a target ratio of 2 [89]. To date, no similar official guidance has been identified from other countries.

Despite the above-mentioned convenience and value, the spot urine Na–K ratio exhibits substantial intraday and interday variability [167,168,170] and serves as an imperfect surrogate for the 24-h urinary Na–K ratio at the individual level (e.g., individual-level correlation: *r* = 0.69) [140]. To mitigate these downsides, multiple spot samples are recommended on different days (e.g., repeated morning/daytime voids across multiple days) [135,172,173], and further validation research is needed to examine its performance across diverse collection protocols.

Importantly, the above limitations should be viewed in context. At the population level, spot urine Na–K ratios show high agreement with 24-h urine Na–K ratios (e.g., population-level correlation *r* = 0.96) [140], supporting their use for surveillance or group comparison. For individual monitoring, although within-day fluctuations exist, repeated measurements taken at a fixed time point (e.g., morning void) may provide meaningful insight into healthy eating. This is because most individuals’ Na–K ratios already deviate substantially from the recommended level (by a factor of 3–5) [26,78,174], and the magnitude of diurnal variation could be negligible compared with this large deviation.

#### Applying Na–K balance to drive individual and industry actions

Pursuing Na–K balance is not merely a matter of reducing sodium and increasing potassium intake—it might be a revolution in traditional cooking and food manufacturing.

Historically, culinary traditions worldwide have emphasized saltiness through the use of salt and high-sodium condiments (e.g., soy sauce, fish sauce), along with processing methods that deplete potassium. For example, blanching, soaking, and discarding cooking water can reduce potassium content by 30%–50% or more [[73], [74], [75]]. These concurrent practices—adding sodium and depleting potassium—underlie the high-Na/low-K profile of prepared foods.

A fundamental shift in culinary practice—applicable to both home-prepared meals and processed foods—involves reducing reliance on salt and appreciating natural food flavors, using alternative lower-sodium flavor enhancers when needed, and preserving or enriching natural potassium content. Specifically, this includes: *1*) reducing the use of salt and high-sodium products; *2*) substituting salt with herbs, spices, garlic, citrus, and vinegar; *3*) adopting cooking or processing methods that retain potassium (e.g., shortening blanching and soaking times, or replacing them with steaming, microwaving, or incorporating pot liquors into soups and stews); *4*) prioritizing minimally processed foods; *5*) increasing the consumption of potassium-rich foods such as legumes, nuts, fruits, and vegetables, which naturally contain 10–100 times more potassium than sodium [175]; and *6*) encouraging and facilitating food manufacturers to redesign workflows to minimize potassium leaching, while also making Na–K ratio or potassium content labeling a mandatory requirement.

From an implementation perspective, education must provide practical, culturally adapted culinary skills targeting individuals, including seasoning with less salt, cooking in ways that retain potassium where possible, and reading labels for both sodium and potassium [23,72]. Policies should incentivize manufacturing changes, such as mandatory Na–K reformulation targets, subsidies for potassium-rich produce, and low Na–K ratio procurement standards for schools and hospitals [23,41,72].

Regarding LSSS: although LSSS (which replace 19%–50% of NaCl with KCl) show effect in lowering blood pressure and reducing cardiovascular disease risk [176,177], it is a partial solution, not a revolution. From a Na–K balance perspective, we prioritize transforming food preparation and dietary behaviors—preparing and enjoying naturally low-Na/K foods, without relying on special substitutes. This aligns with WHO’s core strategy: reducing salt intake, increasing potassium intake from food sources, and conditionally recommending LSSS [23,72,177].

## Discussion

### Summary statement

This narrative review and policy analysis serves as a complement and extension to existing global sodium reduction frameworks. To facilitate strategy development, we developed a novel classification framework that categorizes dietary sodium into natural, formula, and expanded discretionary sources, and further grouped countries into DSD, DFB, and FSD typologies. Our findings confirm that around 80% of the global population resides in DSD nations, where discretionary sodium dominates dietary intake. To strengthen strategies targeting discretionary sources, this study proposes core theoretical principles, a 3-step national implementation roadmap, and 9 multisector action domains to support structured delivery. We further enrich the existing global strategy system by integrating the Na–K balance perspective to guide intervention design and population surveillance. Collectively, our context-specific frameworks and actionable recommendations offer practical pathways enabling countries to tailor interventions to local dominant sodium sources.

### Future research directions

First, apply the proposed or similar framework to evaluate national/regional sodium sources, identify whether discretionary or formula sources predominate, and analyze dietary practices driving high-Na/low-K diets—to guide targeted strategies, health education, and intervention selection. Second, examine which cooking and processing methods (and to what extent) shift the Na–K ratio toward balance, and how to disseminate these techniques. Third, evaluate spot urine Na–K ratio as a surrogate for dietary Na–K balance, and establish protocols for individual and population surveillance. Fourth, promote the Na–K-balance concept in designing and evaluating sodium reduction interventions—beyond the proven but limited use of Na–K self-monitoring devices (2025 review: 8 trials, 1442 subjects) [162]. Fifth, design pragmatic RCTs to assess real-world effectiveness of structurally delivered sodium reduction strategies leveraging existing health systems, regulations, and scalable technologies—moving beyond efficacy trials under ideal conditions.

### Study limitations

First, our country grouping using the novel sodium source framework may have led to misclassification, because many nations report sodium sources by traditional or variable standards, and some lack robust studies. Further research is needed to confirm the categorization. Second, although WHO has outlined sodium reduction policies via the Sodium Country Score Card and provided examples (e.g., food procurement, mass media campaigns) [6], detailed policies addressing discretionary sodium intake may have been unreported and were not identified or cited in this study—primarily due to language barriers and lack of implementation evidence. Ongoing collection and evaluation of such policies are needed, especially those proven to be scalable and successful at the national level. Third, our recommendations, although largely evidence based, may not suit all contexts because of the limited availability of data in some countries. Further refinement is required to approach global guidance.

In conclusion, strategies targeting formula sources remain indispensable globally. For DSD and DFB countries, which are home to 90% of the world’s population, greater emphasis on discretionary sodium interventions is needed. Building on existing frameworks, further progress requires standardized sodium source assessment, solid theoretical foundations, context-specific multisector frameworks for structured implementation, and integration of the Na–K balance principle to guide intervention design and monitoring. Embedding these components into the development and implementation of global and national initiatives will accelerate progress toward WHO sodium reduction targets and improve public health outcomes.

## Author contributions

The authors’ responsibilities were as follows – PZ: conceived the policy review and drafted the first manuscript; FJH, GZ: participated in the literature review and summarization; and all authors: contributed to the revisions and approved the final manuscript.

## Data availability

Data described in the manuscript, codebook, and analytic code will be made available upon request pending application and approval.

## Declaration of generative AI and AI-assisted technologies in the writing process

During the preparation of this work, the authors used DeepSeek in order to check the English grammar. After using this tool/service, the authors reviewed and edited the content as needed and take full responsibility for the content of the publication.

## Funding

This study was supported by the Noncommunicable Chronic Diseases-National Science and Technology Major Project (2026ZD0558000, 2026ZD0558006), which is a grant from the Medical Science and Technology Development Research Center of the National Health Commission of China.

## Conflict of interest

FJH is an unpaid member of Action on Salt and World Action on Salt, Sugar and Health (WASSH). The remaining authors declare that the research was conducted in the absence of any commercial or financial relationships that could be construed as a potential conflict of interest.
